# Case Report: Famotidine for Neuropsychiatric Symptoms in COVID-19

**DOI:** 10.3389/fmed.2020.614393

**Published:** 2020-12-23

**Authors:** Kenneth Alper

**Affiliations:** Departments of Psychiatry and Neurology, NYU Grossman School of Medicine, New York, NY, United States

**Keywords:** COVID-19, SARS-CoV-2, famotidine (FAM), depression, anxiety, psychiatry, cognitive, neuroinflammation

## Abstract

Famotidine is of interest as a possible treatment for COVID-19, with effects on disease-related symptoms and survival reported in observational and retrospective studies, as well as *in silico* predictions of binding to potential SARS-CoV-2 drug targets. Published studies of famotidine for COVID-19 have focused on acute illness, and none have reported on neuropsychiatric symptoms. This case study reports on an 18-year-old man who sought psychiatric treatment for depression and anxiety, disruptive interpersonal conflicts, and impairments in attention and motivation following mildly symptomatic illness with COVID-19. The neuropsychiatric symptoms, which had been present for 16 weeks at the time of the initial evaluation represented a significant departure from the patient's previous behavioral baseline. The patient had no prior psychiatric history preceding his illness with COVID-19, and no history of any prior treatment with psychopharmacological medications. Famotidine 20 mg twice daily administered orally was begun without any additional medications. At 1-week follow-up the patient was much improved. Improvement was sustained through 12 weeks of follow-up during which the patient continued to take famotidine without apparent side effects. With progression of the COVID-19 pandemic it has become evident that persistent disease-related symptoms may follow acute COVID-19 and may include neuropsychiatric symptoms. Controlled clinical research on famotidine for COVID-19 should follow, as well as the development of valid and reliable research diagnostic criteria to define and operationalize the features of a putative COVID-19 neuropsychiatric residual.

## Introduction

Famotidine, a histamine H2 receptor antagonist with labeled indications for heartburn and gastric reflux, has been suggested as a possible treatment for COVID-19 on the basis of observational and retrospective study evidence. In Wuhan, China, in January of this year, retrospective analysis of data of hospitalized patients indicated increased survival in patients with COVID-19 who had been taking famotidine at the time of admission to the hospital. The difference in mortality rates, 14 vs. 27% favoring those who had taken famotidine, did not reach statistical significance (1). Nonetheless, a physician familiar with these data observed an apparent treatment effect within 24 h after his sister, ill with COVID-19, took famotidine.

A published case series reports on 10 outpatients who self-treated with famotidine following the onset of COVID-19 symptoms (2). All of the patients in the series reported marked improvement in disease-related symptoms, with significant improvement in a group mean symptom score evident within 1 day of starting famotidine. Two retrospective cohort studies of patients hospitalized for COVID-19 compared patients who had taken famotidine to those who had not (3, 4). Both studies found a significant reduction of the primary endpoints of death and endotracheal intubation among patients who had taken famotidine, presumably for its labeled indications, within 24 h of hospital admission (3) or within 7 days of COVID-19 screening and/or hospital admission (4). An additional physician-sponsored cohort study of famotidine combined with cetirizine in hospitalized patients found rates of mortality that were said to compare favorably to published inpatient fatality rates from other regions (5).

Published studies of famotidine for the treatment of COVID-19 to date have focused on acute illness, and all but the case series (2) involved inpatients. None of the above studies reported on neuropsychiatric symptoms. With progression of the pandemic, it is increasingly evident that disease-related symptoms, including neuropsychiatric symptoms, may persist after acute illness with COVID-19 (6, 7). This case report describes treatment with famotidine for persistent neuropsychiatric symptoms following acute illness with COVID-19.

## Case Description

An 18-year-old man presented for psychiatric evaluation with complaints of “*I've been anxious, irritated and sad most of the time*…” and “…*inability to get motivated/concentrate and retain information*.” The patient was also seeking a second opinion after a psychotherapist had diagnosed him with Bipolar II Disorder (8).

The patient's presenting psychiatric symptoms were of relatively recent onset, an estimated 16 weeks before the initial evaluation, and represented a distinct change from the patient's previous behavioral baseline. In addition to depressed and anxious mood, he experienced disruptive behavioral episodes with increased emotional reactivity and somatic anxiety symptoms. He described these episodes as “…*break down in tears/hyperventilation. I 'd blow up over something insignificant it would turn into a 5-hour argument*.” These episodes occurred in the context of but were not confined to interpersonal interactions, “*I also suffered them when thinking about career/future prospects*.” Diagnostically, these events had features in common with panic attacks including prominent somatic anxiety with a paroxysmal onset, as well as features evident in bipolar mixed states, including heightened reactivity of mood and irritability. The patient's cognitive complaints included diminished motivation and sustained attention, as well as difficulty recalling memories from the previous days to weeks.

On initial evaluation, the patient scored 16 on the Beck Depression Inventory (BDI) (9) and 17 on the Beck Anxiety Inventory (BAI) (10). He denied any prior history of mood or anxiety symptoms or behavioral changes similar to those that had led him to seek treatment. He had never taken psychiatric medications apart from melatonin for sleep at dosages up to 10 mg per night starting 6 weeks prior to the initial psychiatric evaluation. His entire history of prior psychiatric treatment was limited to three visits with a psychotherapist over the month before the evaluation, which were not regarded to have been of benefit. The patient's family psychiatric history was limited to a paternal half-sibling who the patient viewed as possibly depressed but had never sought treatment. He denied any history suggestive of a substance use disorder and had no history or evidence of psychotic symptoms in the diagnostic interview.

Approximately 19 weeks prior to the initial psychiatric evaluation, in the third week of February 2020, the patient experienced the onset of fatigue and cough (see Table 1). The fatigue persisted for approximately a week and the cough persisted for 3 weeks. The patient did not monitor temperature for 12 days following the onset of the fatigue and cough, as he explained, “*I suspected I just had seasonal allergies/ a cold. When more information about COVID began, including heightened temperature, that is when I began monitoring my temperature*,” and at this point, he was afebrile. At about 16 weeks prior to the evaluation, the fatigue recurred, with the onset at that time of the behavioral changes that would eventually lead the patient to seek psychiatric evaluation.

**Table 1:** Timeline of clinical case history, time is referenced to the initiation of treatment with famotidine (week = 0).

**Event**	**Week**
Probable COVID-19 exposure	−20
Onset of fatigue and cough	−19
Onset of neuropsychiatric symptoms	−16
Positive test for SARS-CoV-2 antibody	−5
Start famotidine	0
Follow-up (weeks +1, 3, 8, 9, 12)	+1–12

The week before the onset of cough and fatigue, the patient, who was attending college outside of New York State, went to an indoor conference in New York City that included approximately 100 attendees. He also visited his family at this time, including his father, a public transit worker in New York City with occupational exposure to the subway system. Both the patient and his father experienced the onset of cough and fatigue the week following their contact with one another, with the father experiencing relatively more severe symptoms. Nether the patient nor his father were tested for SARS-CoV-2 antigen, which was not systematically available in New York at that time. Five weeks prior to the initial psychiatric evaluation, the patient tested positive for SARS-CoV-2 antibody, following his father's positive antibody test result.

The working DSM-5 psychiatric diagnoses were Other Specified Mental Disorder and Mild Neurocognitive Disorder both due to COVID-19, according to a clinical hypothesis that the patient's presenting neuropsychiatric symptoms were related to prior illness with COVID-19 (8). “Other Specified Mental Disorder” indicates that the symptoms did not meet criteria for any specific major mood or anxiety disorder, for example, the patient's disruptive behavioral episodes having some features of both mood and anxiety disorders but not fully meeting the criteria for either. “Mild Neurocognitive Disorder” subsumes symptoms in the domain of executive functions.

The patient was begun on oral famotidine 20 mg twice daily as per the drug labeling. No psychopharmacological or other medications were prescribed, melatonin was discontinued, and no behavioral interventions or lifestyle changes were made. On follow-up a week later, the patient said he felt “much better” and noted substantial improvement regarding his symptoms of heightened emotional reactivity and diminished motivation and sustained attention. He estimated the time interval between starting famotidine and symptomatic improvement at 4 days and described his state on the fourth day as “clear-headed,” “*…I woke up and got out of bed without feeling awful…*.” He reported no side effects. At 3-week follow-up, the patient's BDI and BAI scores were 1 and 2, respectively. A friend familiar with the patient's prior behavioral baseline described the patient's behavioral change following presumed COVID-19 illness and prior to treatment with famotidine as “*…more irritable or quiet…exhausted.”* At 4 weeks following the initiation of famotidine, the friend described, “*… he seemed much more conversational as well as productive…very focused*….” The patient continues to report he is doing well at the time of this writing, 12 weeks following the initiation of treatment with famotidine, which he continues to take at the initially prescribed dosage of 20 mg twice daily.

## Discussion

This case suggests a possible treatment effect of famotidine for persistent neuropsychiatric symptoms following acute illness with COVID-19. It appears generally consistent with observational and retrospective study evidence for an apparent treatment effect of famotidine on disease-related symptoms and survival in COVID-19 (2–5).

The interval of 16 weeks from the initial onset of neuropsychiatric symptoms following apparent illness with COVID-19 to the initiation of treatment with famotidine is a distinctive aspect of this case. Individuals with persistent symptoms following acute illness with COVID-19, the “long haulers,” are an increasing and arguably a presently relatively underserved population. A CDC study found that 35% of adult outpatients who were symptomatic at the time they tested positive for SARS-CoV-2 antigen had not returned to their usual state of health at 2–3 weeks following testing (7). Clinical investigation of famotidine for COVID-19 should include patients with disease-related symptoms at relatively extended time intervals following acute illness.

To date, the author has treated eight other patients in his general psychiatric practice with famotidine for persistent neuropsychiatric symptoms following acute illness with COVID-19. In contrast to the present case, these other patients were already receiving psychopharmacological treatment at the time of onset of COVID-19 and subsequent treatment with famotidine. Within the limits of uncertainty due to the intermingling of factors including psychiatric baseline, emotional responses to the pandemic, and variability in length of treatment with famotidine, most patients appear to have received some benefit. The most frequent domain of symptomatic improvement appears to be “brain fog,” a term applied to a set of symptomatic features suggestive of problems with executive functions, including sustained attention/working memory and motivation, as well as word-finding and short-term memory. Patients have utilized the term “clearer” in their description of a famotidine effect.

Other symptomatic features of a putative COVID-19 neuropsychiatric residual relate to mood, anxiety, and emotional reactivity. Some patients with a prior history of depression describe mood changes following COVID-19 as distinct in quality from their previous depression. The term “despair” has been used, apparently connoting qualities of intensity and hopelessness, which may be of significance regarding suicidal risk. Irritability and highly reactive mood may be evident as interpersonal conflict. The expression of mood and anxiety symptoms may be episodic and paroxysmal. Development of valid and reliable research diagnostic criteria for a putative syndrome of COVID-19 neuropsychiatric residual would provide a basis for defining patient groups for clinical trials and measures of illness severity.

Neuroinflammation plays an increasingly appreciated role in psychiatric disorders (11). SARS-CoV-2 is neuroinvasive and neuroinflammatory (12). Baseline inflammatory markers predicted subsequent anxiety and depression 30 days after discharge from the emergency room or hospital in a cohort study of patients with COVID-19 pneumonia presenting for emergency evaluation (6).

SARS-CoV-2 viral persistence may mediate disease-related symptoms following acute COVID-19 illness. Clinical trial data indicate a potential for persistence of SARs-CoV-2; 41.5% of the subjects in a study of lopinavir–ritonavir still had a viral load detectable by oropharyngeal swab at 28 days following randomization (13), with an additional interval of 13 days between symptom onset and randomization. Further, SARS-CoV-2 may persist in anatomical regions inaccessible to nasal–oropharyngeal swab, such as the gut. SARS-CoV-2 RNA was detectable in fecal samples from 51.8% of COVID-19 patients in a recent meta-analysis (14) and has been reported to persist up to 70 days after symptom onset in individual cases (15). SARS-CoV-2 RNA may continue to be detectable in fecal samples from patients with a negative nasal–oropharyngeal swab and is associated with a longer interval from symptom onset to viral clearance (14, 16–18). Angiotensin-converting enzyme 2, which acts as a host receptor protein to bind coronavirus spikes and enable subsequent viral-host cell membrane fusion and viral entry, is expressed relatively strongly by intestinal epithelial cells (19–22). Future research should investigate a possible association of fecal SARS-CoV-2 RNA with persistent COVID-19 disease-related symptoms.

The SARS-CoV-2 proteins most studied as potential drug targets are the SARS-CoV-2 chymotrypsin-like protease (3CLpro), also known as main protease (Mpro), and SARS-CoV-2 papain-like protease (PLpro). Both of these proteases are critical for viral replication, and PLpro additionally has effects on ubiquitination and interferon that may dysregulate host innate immunity. *In silico* methods, including virtual ligand screening of SARS-CoV-2 proteins against libraries of compounds, predict the binding of famotidine to Mpro (23), PLpro (24, 25), or both (26). These *in silico* predictions await laboratory target validation.

If famotidine is indeed effective for the treatment of COVID-19, it might be hypothesized to act as a virustatic protease inhibitor, possibly on the basis of an interaction with proteins involved in viral replication, such as but not limited to Mpro or PLpro. This may suggest a general analogy to virustatic protease inhibitors such as those used to treat HIV or hepatitis C (27). An alternative hypothesis suggests that the therapeutic effect of famotidine may be due to its action as an H2 antagonist against inflammatory effects mediated by H2-related signaling in the presence of a highly inflammatory pathogen (5).

The effect of famotidine appears rapid in its onset; in this present case, the patient reported significant improvement at 4 days. In the case series of 10 outpatients who self-treated acute COVID-19 illness with famotidine (2), group mean symptom scores separated significantly from pretreatment baseline by day 1 of treatment. These patients who self-treated with famotidine utilized dosages ranging from 60 to 240 mg daily for a median duration of 11 days. In the two studies that compared groups of hospitalized patients on the basis of famotidine use, the respective median values for the total cumulative dose received are 136 mg and 80 mg, and 5.8 and 4 days for duration of treatment (3, 4). Treatment in this present case is ongoing at 12 weeks. The risk–benefit calculus would appear to favor caution in lowering and discontinuing famotidine. Factors that favor continuing famotidine are its safety and the possibility that if the drug is indeed effective, it could be providing extended suppressive therapy in a setting of months of previous symptomatic illness. Discussion with the patient regarding when to initiate a gradual taper of famotidine is ongoing as of this writing.

A limitation of a single case report is its unknown reproducibility and the need for confirmation by controlled clinical investigation. Even if famotidine has indeed had a treatment effect in this case, its generalizability may be limited in view of the patient's relatively young age, which may have been a factor in his apparently favorable response and may not be representative of older people with COVID-19. The patient's use of melatonin might be considered a possible confound in view of the suggestion that its antioxidant actions might have beneficial effects on pulmonary inflammation (28). However, the patient commenced and stopped the use of melatonin without a change in clinical status, in contrast to the close temporal correspondence of treatment with famotidine and clinical improvement.

The premise that the patient's behavioral symptoms are etiologically related to COVID-19 requires their occurrence to have been subsequent to, and not prior to COVID-19. Testing for SARS-CoV-2 antigen was not obtained in this case. However, in New York City in late February of 2020, a diagnosis of COVID-19 would have been clinically likely for two individuals with new onset fatigue and cough a week following their contact with one another, both of whom subsequently tested positive for SAR-CoV-2 antibody. The patient's presenting neuropsychiatric symptoms began ~3 weeks following the apparent onset of COVID-19 (see Table 1).

The possibility of a placebo effect potentially confounds the attribution of clinical improvement to famotidine. Attribution of the apparent clinical response to a placebo effect in this case would imply suggestion as the basis for the presenting neuropsychiatric symptoms, and weigh against mediation by biological effects of SARS-CoV-2 viral infection. There does not appear to be a compelling psychological explanation for the appearance of neuropsychiatric symptoms that departed markedly from the patient's prior behavioral baseline. Nonetheless, the possibility of a placebo effect is structural to any individual psychiatric case report due to reliance on behavioral features and lack of biological markers in present psychiatric diagnosis.

It is possible that this case report and other observational and retrospective evidence for an effect of famotidine on disease-related symptoms in COVID-19 might represent a collective type 1 statistical error, a false positive. The cost of this type of error would be the expense of a controlled clinical research effort that fails to confirm the hypothesized treatment effect. However, such a clinical research effort may be justified. A type 2 error, a false negative, is potentially more costly. Famotidine is inexpensive, and relatively safe. If it is indeed effective as an antiviral against SARS-CoV-2, famotidine may provide a novel mechanism of action for potential polytherapy synergies or offsetting antiviral resistance, as well as a scaffold for drug design involving rational pharmaceutical synthesis of structural analogs informed by structure activity relationships.

## Conclusion

This case report is generally consistent with observational and retrospective evidence for an apparent effect of famotidine on disease-related symptoms and survival in COVID-19 (2–5). It should be followed by controlled clinical investigation. There is a need to develop treatment approaches for residual disease-related symptoms, including neuropsychiatric symptoms weeks to months following acute illness with COVID-19. Clinical research on famotidine for COVID-19 should include assessment of neuropsychiatric symptoms and more extended intervals of follow-up. This work would be optimally enabled by the development of valid and reliable research diagnostic criteria to define and operationalize the features of a putative syndrome of COVID-19 neuropsychiatric residual.

## Data Availability Statement

The original contributions presented in the study are included in the article, further inquiries can be directed to the author.

## Ethics Statement

Ethical review and approval was not required for the study on human participants in accordance with the local legislation and institutional requirements. The patients/participants provided their written informed consent to participate in this study. Written informed consent was obtained from the individual(s) for the publication of any potentially identifiable images or data included in this article.

## Author Contributions

KA evaluated and treated the patient as described in this case report and authored the manuscript.

## Conflict of Interest

The author declares that the research was conducted in the absence of any commercial or financial relationships that could be construed as a potential conflict of interest.

## References

[1] Borrell B New York Clinical Trial Quietly Tests Heartburn Remedy Against Coronavirus. Sciencemag.org (2020). Available online at: https://www.sciencemag.org/news/2020/04/new-york-clinical-trial-quietly-tests-heartburn-remedy-against-coronavirus (accessed August 30, 2020).

[2] Janowitz T Gablenz E Pattinson D Wang TC Conigliaro J Tracey K. Famotidine use and quantitative symptom tracking for COVID-19 in non-hospitalised patients: a case series. Gut. (2020) 69:1592–7. 10.1136/gutjnl-2020-321852 32499303 PMC7299656

[3] Freedberg DE Conigliaro J Wang TC Tracey KJ Callahan MV Abrams JA. Famotidine use is associated with improved clinical outcomes in hospitalized COVID-19 patients: a propensity score matched retrospective cohort study. Gastroenterology. (2020) 159:1129–31.e3 10.1053/j.gastro.2020.05.053 32446698 PMC7242191

[4] Mather JF Seip RL Mckay RG. Impact of famotidine use on clinical outcomes of hospitalized patients with COVID-19. Am J Gastroenterol. (2020) 115:1617–23. 10.14309/ajg.0000000000000832 32852338 PMC7473796

[5] Hogan RB II Hogan RB III Cannon T Rappai M Studdard J Paul D Dooley TP. Dual-histamine receptor blockade with cetirizine - famotidine reduces pulmonary symptoms in COVID-19 patients. Pulm Pharmacol Ther. (2020) 63:101942. 10.1016/j.pupt.2020.101942 32871242 PMC7455799

[6] Mazza MG De Lorenzo R Conte C Poletti S Vai B Bollettini I. Anxiety and depression in COVID-19 survivors: role of inflammatory and clinical predictors. Brain Behav Immun. (2020) 89:594–600. 10.1016/j.bbi.2020.07.037 32738287 PMC7390748

[7] Tenforde MW Kim SS Lindsell CJ Billig Rose E Shapiro NI Files DC. Symptom duration and risk factors for delayed return to usual health among outpatients with COVID-19 in a multistate health care systems network - United States, March-June 2020. Morb Mortal Wkly Rep. (2020) 69:993–8. 10.15585/mmwr.mm6930e1 32730238 PMC7392393

[8] American Psychiatric Association Diagnostic and Statistical Manual of Mental Disorders, Fifth Edition Washington, DC: American Psychiatric Association (2013).

[9] Beck AT Erbaugh J Ward CH Mock J Mendelsohn M. An inventory for measuring depression. Arch Gen Psychiatry. (1961) 4:561–71. 10.1001/archpsyc.1961.01710120031004 13688369

[10] Fydrich T Dowdall D Chambless DL Reliability and validity of the beck anxiety inventory. J Anxiety Disord. (1992) 6:55–61. 10.1016/0887-6185(92)90026-4

[11] Najjar S Pearlman DM Alper K Najjar A Devinsky O Neuroinflammation and psychiatric illness. J Neuroinflammation. (2013) 10:43 10.1186/1742-2094-10-43 23547920 PMC3626880

[12] Cataldi M Pignataro G Taglialatela M. Neurobiology of coronaviruses: potential relevance for COVID-19. Neurobiol Dis. (2020) 143:105007. 10.1016/j.nbd.2020.105007 32622086 PMC7329662

[13] Cao B Wang Y Wen D Liu W Wang J Fan G. A trial of lopinavir–ritonavir in adults hospitalized with severe covid-19. N Engl J Med. (2020) 382:1787–99. 10.1056/NEJMoa2001282 32187464 PMC7121492

[14] Van Doorn AS Meijer B Frampton CMA Barclay ML De Boer NKH. Systematic review with meta-analysis: SARS-CoV-2 stool testing and the potential for faecal-oral transmission. Aliment Pharmacol Ther. (2020) 52:1276–88. 10.1111/apt.16036 32852082 PMC7461227

[15] Hua CZ Miao ZP Zheng JS Huang Q Sun QF Lu HP. Epidemiological features and viral shedding in children with SARS-CoV-2 infection. J Med Virol. (2020) 92:2804–12. 10.1002/jmv.26180 32542750 PMC7323101

[16] Han C Duan C Zhang S Spiegel B Shi H Wang W Digestive symptoms in COVID-19 patients with mild disease severity: clinical presentation, stool viral RNA testing, and outcomes. Am J Gastroenterol. (2020) 115:916–23. 10.14309/ajg.0000000000000664 32301761 PMC7172493

[17] Ling Y Xu SB Lin YX Tian D Zhu ZQ Dai FH. Persistence and clearance of viral RNA in 2019 novel coronavirus disease rehabilitation patients. Chin Med J. (2020) 133:1039–43. 10.1097/CM9.0000000000000774 32118639 PMC7147278

[18] Wang XM Zheng JW Guo L Yao H Wang LY Xia XD. Fecal viral shedding in COVID-19 patients: clinical significance, viral load dynamics and survival analysis. Virus Res. (2020) 289:7. 10.1016/j.virusres.2020.198147 32866537 PMC7455175

[19] Du M Cai G Chen F Christiani DC Zhang Z Wang M. Multiomics evaluation of gastrointestinal and other clinical characteristics of COVID-19. Gastroenterology. (2020) 158:2298–301 e7. 10.1053/j.gastro.2020.03.045 32234303 PMC7270476

[20] Lee JJ Kopetz S Vilar E Shen JP Chen K Maitra A. Relative abundance of SARS-CoV-2 entry genes in the enterocytes of the lower gastrointestinal tract. Genes. (2020) 11:645. 10.3390/genes11060645 32545271 PMC7349178

[21] Qi F Qian S Zhang S Zhang Z. Single cell RNA sequencing of 13 human tissues identify cell types and receptors of human coronaviruses. Biochem Biophys Res Commun. (2020) 526:135–40. 10.1016/j.bbrc.2020.03.044 32199615 PMC7156119

[22] Venkatakrishnan AJ Puranik A Anand A Zemmour D Yao X Wu X. Knowledge synthesis of 100 million biomedical documents augments the deep expression profiling of coronavirus receptors. Elife. (2020) 9:e58040. 10.7554/eLife.58040.sa2 32463365 PMC7371427

[23] Wu C Liu Y Yang Y Zhang P Zhong W Wang Y. Analysis of therapeutic targets for SARS-CoV-2 and discovery of potential drugs by computational methods. Acta Pharm Sin B. (2020) 10:766–88. 10.1016/j.apsb.2020.02.008 32292689 PMC7102550

[24] Kandeel M Abdelrahman AHM Oh-Hashi K Ibrahim A Venugopala KN Morsy MA. Repurposing of FDA-approved antivirals, antibiotics, anthelmintics, antioxidants, and cell protectives against SARS-CoV-2 papain-like protease. J Biomol Struct Dyn. (2020). 10.1080/07391102.2020.1784291. [Epub ahead of print]. PMC7332862 32597315

[25] Sen Gupta PS Biswal S Singha D Rana MK. Binding insight of clinically oriented drug famotidine with the identified potential target of SARS-CoV-2. J Biomol Struct Dyn. (2020). 10.1080/07391102.2020.1784795. [Epub ahead of print]. 32579065

[26] Ortega JT Serrano ML Jastrzebska B. Class A G protein-coupled receptor antagonist famotidine as a therapeutic alternative against SARS-CoV2: an *in silico* analysis. Biomolecules. (2020) 10:954. 10.3390/biom10060954 32599963 PMC7355875

[27] Stahlmann R Lode H. Medication for COVID-19-an overview of approaches currently under study. Dtsch Arztebl Int. (2020) 117:213–9. 10.3238/arztebl.2020.0213 32343658 PMC7196844

[28] Shneider A Kudriavtsev A Vakhrusheva A. Can melatonin reduce the severity of COVID-19 pandemic? Int Rev Immunol. (2020) 39:153–62. 10.1080/08830185.2020.1756284 32347747

